# Stability and infectivity of novel pandemic influenza A (H1N1) virus in blood-derived matrices under different storage conditions

**DOI:** 10.1186/1471-2334-11-354

**Published:** 2011-12-22

**Authors:** Xue Wang, Olga Zoueva, Jiangqin Zhao, Zhiping Ye, Indira Hewlett

**Affiliations:** 1 Laboratory of Molecular Virology, Division of Emerging and Transfusion Transmitted Diseases, CBER/FDA, Building 29B, Rm 4NN22 8800 Rockville Pike, Bethesda, MD 20892, USA; 2 Division of Viral Products, Center for Biologics Evaluation and Research, Food and Drug Administration, Bethesda, MD 20892, USA

## Abstract

**Background:**

Influenza A virus has been detected in the blood of some infected individuals, and may pose a safety concern for collection, handling and transport of specimens for epidemiological and public health investigations if infectious virus is present in samples. Furthermore the effect of storage on virus stability and infectivity has not been well studied.

**Methods:**

We examined the stability of novel pandemic influenza A (H1N1) virus RNA when the virus was stored in phosphate buffered saline (PBS), plasma, or buffy coated blood at either room temperature or 4°C using a sensitive Taqman RT-PCR assay. We also investigated virus infectivity using the EID_50 _assay when virus was stored in PBS, plasma, or buffy coats isolated from blood at 4°C.

**Results:**

Viral RNA stability was affected by the matrix used for storage. The recovery of viral RNA was highest when virus was stored in PBS with lower amounts being recovered from plasma and buffy coats at either room temperature or 4°C. Incubation time did not appear to be a major factor for viral RNA stability, although there was gradual decline after longer periods post-incubation. Both sample matrix and incubation time affected virus infectivity. The decay in virus infectivity was greatest in PBS followed by buffy coats and plasma. Virus infectivity was abolished in buffy coats at day 20 post-incubation when virus concentrations were low.

**Conclusion:**

These data indicate that encapsidated viral RNA was stable overall in all three liquid matrices at room temperature or 4°C although it was most stable in PBS; virus infectivity in buffy coats at 4°C decayed in a time dependent manner while it remained unchanged in plasma. These findings have implications for storage, handling and transport of blood derived samples from influenza patients for epidemiological and laboratory investigations. It should be noted that there is little known about influenza viremia, and whether influenza viruses can be transmitted by blood or blood derived samples.

## Background

Influenza is a major cause of morbidity and mortality in the United States and worldwide. The possibility of an influenza pandemic has focused attention on the epidemiology and pathophysiology of influenza, including its potential for viremia in the acute phase of infection [1].

Influenza A viruses belong to the *Orthomyxoviridae *family of RNA viruses. They contain eight segments of negative sense RNA [2]. In April 2009, a novel pandemic influenza A (H1N1) virus was identified in Mexico and the United States [3]. Thus far, pandemic H1N1 has been detected in more than 200 countries and the strain has caused more than 17,000 deaths [4].

Early in the 1960s, viremia was found in patients infected with influenza A virus in Asia [5,6]. Since then, other groups have reported similar findings [6-9]. It has also been reported that viral RNA could be detected in the blood of humans with fatal outcomes while no viral RNA could be detected in the blood of surviving H5N1-infected individuals [10]. Recently, it has been reported that pandemic influenza H1N1 RNA was detected in 14/139 patients included in a study, by RT-PCR, during May 2009 - April 2010 in Hong Kong [11]

In addition to nasal secretions, buffy coat prepared from blood samples are often used for epidemiological and public health investigations. The likelihood that virus may be present in these samples could be a safety concern for collection, handling and transport of specimens. Additionally, if stored specimens are used for viremia determinations, there is a need to know how storage conditions affect virus stability in order to define conditions that would adversely affect accuracy of test results obtained using such samples. Under normal conditions, whole blood is collected, stored at 2 ~ 8°C, or processed within 6 h of collection. Although it is always advantageous to use or process blood as soon as possible, most of the time it may be stored for weeks, or shipped to another area for testing. In order to evaluate virus stability and infectivity in buffy coats, we spiked varying amounts of pandemic influenza A (H1N1) virus in PBS, and blood derived buffy coat or plasma and held samples under different temperature conditions. The samples were then assayed using the Taqman PCR and HA activity assay to determine effects on stability and infectivity respectively.

## Materials and methods

### Virus

A novel pandemic influenza A (H1N1) virus stock, A/California/04/2009 was obtained from the Centers for Disease Control and Prevention (CDC, Atlanta, GA). The virus was propagated in 9-11 day embryonic hen's eggs. The propagated virus was maintained at -80°C until use in the study.

### Determination of viral titers

Embryonated 11 day old embryonic hen's eggs were evaluated for viability and spray disinfected. The virus was serially diluted from 10^-1 ^to 10^-8 ^in PBS containing 10 μg/ml gentamicin and 0.1 ml was injected into the allantoic cavity of each of five eggs per dilution. Eggs were incubated at 33°C for 3 days before chilling for 8 h at 4°C. Allantoic fluids were collected from each egg and 50 μl tested for hemagglutination with turkey erythrocytes (CBT Farms, Chestertown, MD). The Kärber formula was used to calculate the EID_50 _for each strain [12]. Data were expressed as 50% egg infectious dose (EID_50_) per milliliter.

### Preparation of virus dilutions in PBS, Buffy coats or plasma

10 μl of 3.55 × 10^8 ^EID_50_/ml virus stock was diluted to 3.55 × 10^6^, 3.55 × 10^5^, or 3.55 × 10^4 ^EID_50 _in 10 μl of phosphate buffered saline (PBS). The dilutions above were mixed with 130 μl of PBS, human plasma or buffy coat (from the NIH Buffy coats Center) at either room temperature or at 4°C for different periods of time as indicated, and tested for virus stability using real-time PCR and infectivity using the HA activity assay described above. Virus stability and infectivity for time "0" indicated in the figures was tested immediately (within 10 min) after mixture of virus and the specific matrix.

### Real-time PCR

Quantitative real-time reverse-transcriptase (RT) PCR was also used for quantitation of virus in PBS, plasma or buffy coats (a total of 140 μl). Viral nucleic acids were isolated using the QIAamp Viral RNA Mini Kit (Valencia, CA 91355) according to the manufacturer's protocol. We designed a set of primers and probes for the matrix gene, M, of the novel H1N1 influenza A virus, according to the GenBank database. The forward primer was 5'-CGTCAGGCCCCCTCAAA-3', and the reverse primer was 5'- TTTCCTGCAAAGACACTTTCCA-3'. The TaqMan probe was oligonucleotide 5'- CGAGATCGCGCAGAGA-3', coupled with a reporter dye [6-carboxy fluorescein] (FAM) at the 5' end, a non-fluorescent quencher and a minor groove binder (MGB), that served as a Tm enhancer, at the 3' end. The nucleic acids were amplified and detected in an automated TaqMan 7500 Analyzer by using the QuantiTect™ Probe RT-PCR kit (Qiagen Inc., Valencia, CA). The 25-μl PCR mixture consisted of 100 nM each of primers and 100 nM probe. Following three thermal steps at 55°C for 5 min, 50°C for 30 min and 95°C for 10 min, 45 cycles of two-step PCR at 95°C for 15 s and at 60°C for 1 min were performed. The limit of detection was 1 fg of virus RNA per reaction with the TaqMan assay since the initial sample dilution was 1:10 (for detail see Additional file 1). The data were represented as an average of triple experiments.

### Statistical analysis

The unpaired Student's *t *test was used for data analyses as indicated, and a value of *p *< 0.05 was considered significant (*) and *p *< 0.01 (**) very significant.

## Results

### Viral RNA detection in virus spiked PBS, plasma or Buffy coat held at room temperature up to 72 h

**Figure 1:**
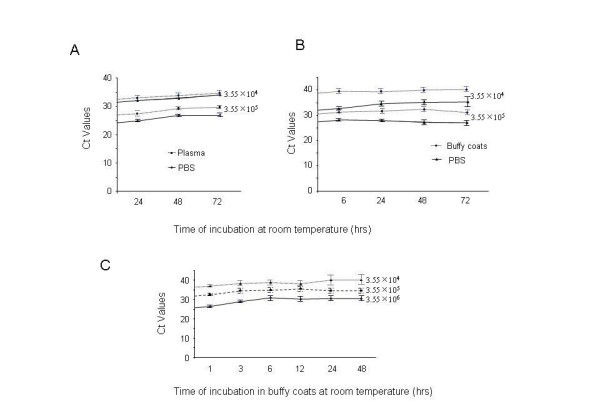
**Stability of influenza RNA at room temperature in different matrices at different spiking concentrations**. 3.55 × 10^6^, 3.55 × 10^5^, or 3.55 × 10^4 ^EID_50 _of H1N1 virus in 10 μl of PBS, diluted as stated in Materials and Methods was mixed with 130 μl of PBS, plasma, or buffy coats for different periods of time indicated. Real-time RT-PCR was performed with TaqMan and, the results were shown as Ct value in RT-PCR. (**A**). Comparison of RNA recovery from samples with different virus concentrations stored in PBS vs. in plasma. (**B**). Comparison of RNA recovery from different virus concentrations stored in PBS vs. buffy coats. (**C**). Comparison of RNA yield from different virus concentrations stored in buffy coats.

Ten fold dilutions of H1N1 virus in 10 μl of PBS starting with 3.55 × 10^6 ^to 3.55 × 10^4 ^EID_50 _of H1N1 virus in 10 μl of PBS were mixed with 130 μl of PBS, plasma, or buffy coat separately and held for different periods of time at room temperature. Nucleic acid was isolated and quantitated using the TaqMan RT-PCR assay. As shown in Figure 1, higher amounts of viral RNA were recovered from PBS relative to plasma or buffy coats, but no significant changes were found within the group of PBS, plasma, or buffy coats up to 72 h, suggesting that there is no significant H1N1 influenza RNA degradation under these conditions. We also found that viral RNA detection was greater with higher titers of virus input in buffy coats, and there was no significant change of viral RNA within 48 h of storage in buffy coats (Figure 1C).

### Viral RNA recovery in Buffy coats stored at 4°C

**Figure 2:**
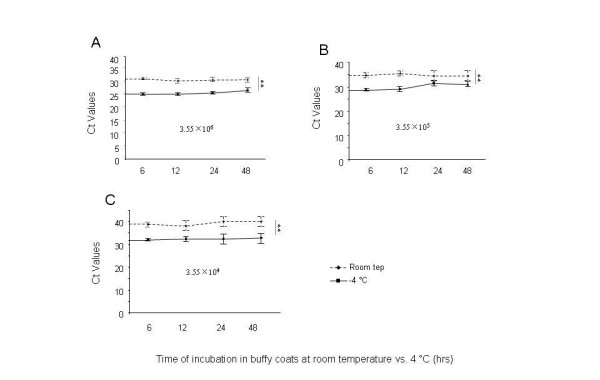
**Stability of influenza RNA at room temperature and 4 C when spiked into buffy coats at different concentrations**. 3.55 × 10^6 ^(**A**), 3.55 × 10^5 ^(**B**), or 3.55 × 10^4 ^(**C**) EID_50 _of H1N1 virus in 10 μl of PBS, diluted as stated in Materials and Methods was mixed with 130 μl of buffy-coated buffy coats for different periods of time indicated. Real-time RT-PCR was performed with TaqMan, and the results were shown as Ct value in RT-PCR.

Ten fold dilutions of H1N1 virus as described above were added to 130 μl of buffy coats and stored at room temperature, or 4 C for up to 48 h to test whether the viral RNA is degraded in buffy coats at different incubation temperatures. As shown in Figure 2, viral RNA yield was significantly increased at 4 C relative to room temperature for three different concentrations of virus incubated, although the copy number of viral RNA in buffy coats was not significantly changed all the time up to 48 h within each group of either room temperature or 4°C (Figure 1A, B or 1C).

### No significant change in viral RNA yield within the group of PBS, plasma or Buffy coats at 4°C for up to 40 days

**Figure 3:**
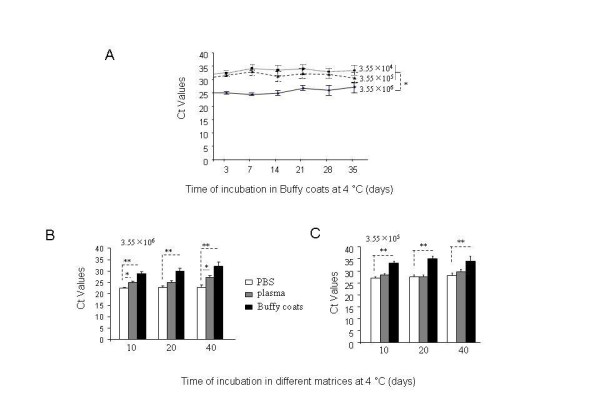
**Stability of influenza RNA at 4 C in different matrices at different spiking concentrations**. 3.55 × 10^6^, 3.55 × 10^5^, or 3.55 × 10^4 ^EID_50 _of H1N1 virus in 10 μl of PBS diluted as stated in Materials and Methods was mixed with 130 μl of PBS, plasma, or buffy coats for different periods of time indicated. Real-time RT-PCR was performed using TaqMan, the results were shown as Ct value in RT-PCR. (**A**). Comparison of RNA recovery from different concentrations of virus stored in buffy coats. (**B**). Comparison of RNA recovery from 10 μl of 3.55 × 10^6 ^EID_50 _of H1N1 virus stored in PBS, plasma, or buffy coats. (**C**). Comparison of RNA recovery from 10 μl of 3.55 × 10^5 ^EID_50 _of H1N1 virus stored in PBS, plasma, or buffy coats.

To study the recovery of viral RNA stored in buffy coats at 4°C for different periods of time, H1N1 virus at the concentrations noted above was mixed with 130 μl of buffy coats, incubated 4°C for different periods of time. After isolation of nucleic acid and viral RNA detection with TaqMan RT-PCR assay, viral RNA copy number was not significantly changed during the incubation period at 4°C for all concentrations tested (Figure 3A).

We then compared viral RNA stability after incubation in either PBS, plasma, or buffy coats in the presence of 3.55 × 10^6^, or 3.55 × 10^5 ^EID_50 _of H1N1 virus at 4°C, and found a similar trend in that there was no significant change of RNA recovery within each group of PBS, plasma or buffy coats (Figure 3B &3C), although there was gradual decline after a longer post-incubation period (Figure 3B). These data suggest that incubation time is not a major factor affecting viral RNA quantitation, using RT-PCR, and TaqMan assays to detect H1N1 viral RNA.

### Virus infectivity in PBS, plasma or Buffy coats at 4°C

**Figure 4:**
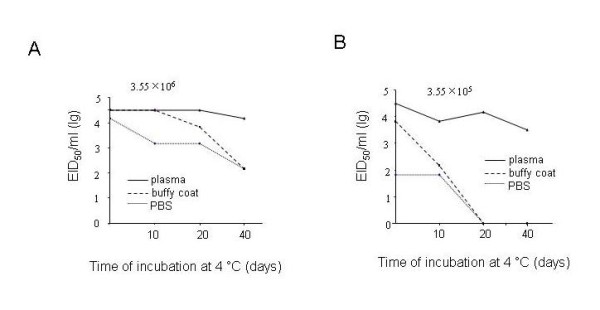
**Stability of influenza infectivity at 4 C in different matrices at different spiking concentrations**. 10 μl of 3.55 × 10^6 ^EID_50 _of H1N1 virus (**A**), or 10 μl of 3.55 × 10^5 ^EID_50 _of H1N1 virus (**B**) diluted as stated in Materials and Methods was mixed with 130 μl of PBS, plasma, or buffy coats for different periods of time indicated. EID50 assay were performed and data were shown as log 10 (EID_50_)/ml.

Given that viral RNA copy number was not significantly affected during different periods of time, and that viral RNA recovery was in the following order: PBS > plasma > buffy coats, we further determined whether virus infectivity was affected after incubation in PBS, plasma, or buffy coats. 10 μl of PBS containing 3.55 × 10^6^, or 3.55 × 10^5 ^EID_50 _of H1N1 virus was incubated in PBS, plasma, or buffy coats at 4°C for different periods of time and EID_50 _assay was performed. We found increased loss of infectivity when virus was stored in PBS relative to buffy coats, or plasma for both virus concentrations (Figure 4A &4B); virus infectivity was less affected by storage in plasma, although there was a gradual decline at lower virus concentration (3.55 × 10^5 ^EID_50 _of H1N1 virus) using the EID_50 _assay (Figure 4B). At higher concentrations (3.55 × 10^6 ^EID_50 _of H1N1 virus), virus infectivity was significantly abolished in PBS at day 10 postincubation relative to plasma and buffy coats (Figure 4A); and loss of virus infectivity was slower in buffy coats relative to PBS but higher than in plasma (Figure 3A). Although infectivity loss at lower concentrations (3.55 × 10^5 ^EID_50 _of H1N1 virus) in PBS, or in buffy coats was reduced to zero at day 20 postincubation, the loss of virus infectivity in buffy coats was slower than in PBS at 10 day postincubation; and virus infectivity declined only gradually when stored in plasma, but was not abolished even at 40 day post-incubation (Figure 4B). Therefore, loss of virus infectivity was highest in PBS followed by buffy coats and plasma. Virus in plasma showed no significant loss in infectivity.

## Discussion

The possibility of an influenza pandemic has focused attention on the epidemiology and pathophysiology of influenza, with specific emphasis on strains of pandemic potential. Viremia has been reported in some patients infected with influenza A virus [5-9,11]. Therefore, stability and infectivity of influenza virus in blood samples during collection, processing and transport is of potential concern. The novel pandemic influenza A (H1N1) virus was used to study the effect of various storage conditions on the ability to detect influenza A viral RNA and virus infectivity in buffy coats relative to PBS and plasma, because the H1N1 virus has been found almost everywhere in the world [4].

Currently, influenza virus is detected and characterized by the use of real-time RT-PCR assays [13]. Virus culture is often used to confirm the presence of infectious virus in clinical samples. Rapid influenza antigen tests have also been used to detect virus, although their specificity has hampered widespread for influenza diagnosis [14]. Our study focused on the use of RT-PCR and infectivity as a measure of influenza virus stability in the blood matrices. The use of an RT-PCR assay directed at conserved regions of the influenza virus matrix gene [15] has proved invaluable as a frontline screening assay that delivers rapid, specific results. Following identification of an influenza virus positive result for the matrix gene sequences, the identification of potential viral HA gene subtypes and determination of their subtype is carried out using a combination of additional RT-PCR assays and sequence analysis [13]. In our study, we used an RT-PCR assay to study the stability of pandemic H1N1 RNA, when stored in PBS, plasma, or buffy coats. Our results showed that the H1N1 RNA copy numbers in PBS, buffy coats, or plasma maintained for up to 3 days storage (at room temperature) or up to 35 days storage (at 4 C) was relatively stable, and showed no significant change. (Figure 1, 3), suggesting that storage time under these conditions may not be a major issue for viral RNA degradation. However, we found that RNA copy number declined more significantly in buffy coats at room temperature rather than at 4 C (Figure 2). These findings could be taken into consideration when evaluating virus infectivity or stability in blood matrices such as buffy coats or plasma of infected persons.

Viral RNA copy numbers were significantly lower in buffy coats relative to plasma, or PBS under our experimental conditions and one possible reason may be due to virus adsorption to blood components such as platelets and red cells [16]. Higher temperature may cause more virions to associate with these blood derived components [7]. Therefore, more viral RNA was recovered and detected in PBS. Further study will be needed to examine the effect of different anticoagulants in blood collection, such as EDTA, heparin and sodium citrate, on viral RNA recovery.

Although the RT-PCR assay is a useful tool to identify influenza viral RNA stability, full characterization of virus infectivity using an EID_50 _assay is necessary to establish the effects of storage on influenza viruses. Our experiments showed that virus infectivity decays at the time-dependent manner, although viral RNA copy number was relatively stable during the experimental period. Proteins binding to viral RNA, which are required for infection of host cells, might be more prone to disassociation from each other or from viral RNA in PBS relative to plasma, and they may be easily bound to the fibrin network as well which decreases their recovery (Figure 3). For these reasons, at lower concentrations of virus, there may be a higher likelihood for significant loss of infectivity over time (comparison of Figure 4A with 4B).

## Conclusion

We have assessed the stability of novel pandemic influenza A (H1N1) virus in PBS, plasma and buffy coats subjected to conditions often encountered in specimen handling, transport, and storage. We found that the RNA copy number did not change significantly when stored in PBS, buffy coats or plasma at room temperature up to 72 h or 4°C for up to 40 days. Although the recovery of viral RNA copy number was greater in PBS followed by plasma and buffy coats at either room temperature or 4°C, loss of virus infectivity was higher in PBS compared with buffy coats; infectivity in plasma did not change significantly under these conditions over time, especially at high doses of virus. We observed good correlation between H1N1 viral load and infectivity loss in PBS, plasma or buffy coats using the EID_50 _assay. In conclusion, the findings of this study indicate that conditions of storage of blood plasma and buffy coats, including temperature and length of time could have an impact on influenza virus infectivity in these matrices and should be taken into consideration in handling and transport of samples for diagnostic testing and epidemiology studies.

## Competing interests

The authors declare that they have no competing interests.

## Authors' contributions

XW participated in the design, data collection and analysis, and drafted the manuscript.

OZ and JZ participated in the data collection. ZY participated in the analysis and commented on drafts of the manuscript. IH conceived of the project, participated in the design and drafted the manuscript. All authors approved the final manuscript.

## Pre-publication history

The pre-publication history for this paper can be accessed here:

http://www.biomedcentral.com/1471-2334/11/354/prepub

## Supplementary Material

Additional file 1 **Supplement**. Amplification curve for A (H1N1) influenza virus real-time RT-PCR assay. Shown are the serial dilutions of A/California/04/2009 (H1N1) RNA (final concentrations 1 fg viral RNA per reaction) with the FAM (novel swine-origin) probe. Reactions were run in duplicate. The forward primer is 5'-CGTCAGGCCCCCTCAAA-3', the reverse primer is 5'- TTTCCTGCAAAGACACTTTCCA-3', and the probe is 5'- CGAGATCGCGCAGAGA-3'. Click here for file
