# Supplementary figures and images for: Stability and infectivity of novel pandemic influenza A (H1N1) virus in blood-derived matrices under different storage conditions
Source: BMC Infect Dis. 2011 Dec 22;11:354. doi: 10.1186/1471-2334-11-354 (PMC3260324; doi:10.1186/1471-2334-11-354)

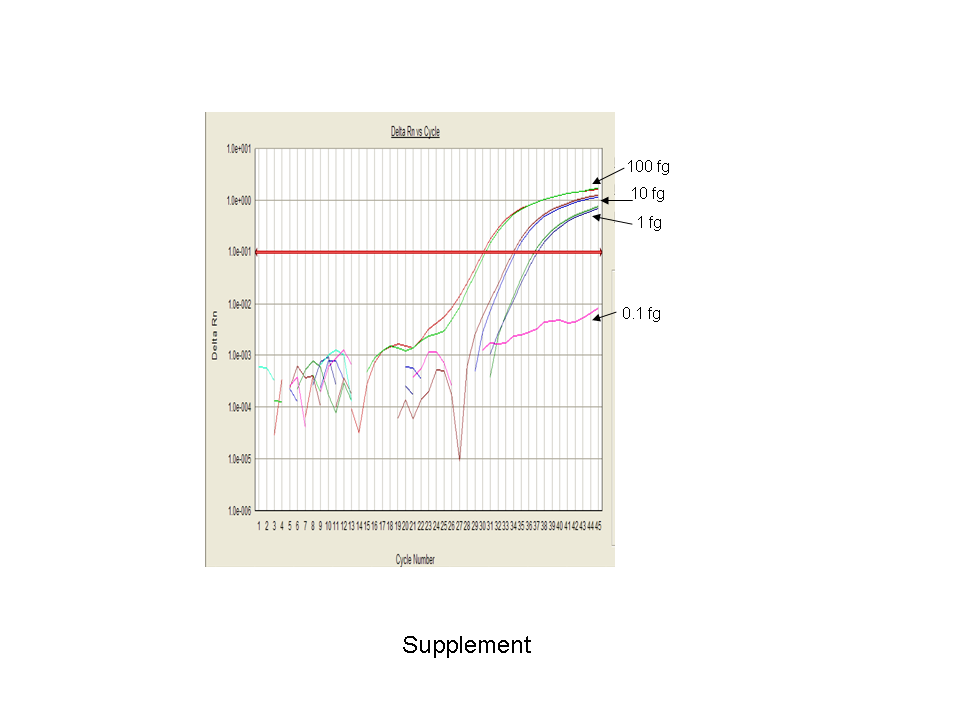

Supplement: Additional file 1 — Supplement. Amplification curve for A (H1N1) influenza virus real-time RT-PCR assay. Shown are the serial dilutions of A/California/04/2009 (H1N1) RNA (final concentrations 1 fg viral RNA per reaction) with the FAM (novel swine-origin) probe. Reactions were run in duplicate. The forward primer is 5'-CGTCAGGCCCCCTCAAA-3', the reverse primer is 5'- TTTCCTGCAAAGACACTTTCCA-3', and the probe is 5'- CGAGATCGCGCAGAGA-3'. [file 1471-2334-11-354-S1.TIFF]
